# Comparing Reminders Sent via SMS Text Messaging and Email for Improving Adherence to an Electronic Health Program: Randomized Controlled Trial

**DOI:** 10.2196/31040

**Published:** 2022-03-18

**Authors:** Adam Kulhánek, Katerina Lukavska, Roman Gabrhelík, Daniel Novák, Václav Burda, Jindřich Prokop, Marianne T S Holter, Håvar Brendryen

**Affiliations:** 1 Department of Addictology General University Hospital in Prague Prague Czech Republic; 2 Department of Addictology, First Faculty of Medicine Charles University Prague Czech Republic; 3 Department of Psychology, Faculty of Education Charles University Prague Czech Republic; 4 Department of Cybernetics, Faculty of Electrical Engineering Czech Technical University in Prague Prague Czech Republic; 5 The Norwegian Centre for Addiction Research (SERAF), Faculty of Medicine University of Oslo Oslo Norway; 6 Department of Psychology, Faculty of Social Sciences University of Oslo Oslo Norway

**Keywords:** eHealth, randomized controlled trial, adherence, reminders, SMS text messaging, email, smoking cessation, text message

## Abstract

**Background:**

eHealth interventions can help people change behavior (eg, quit smoking). Reminders sent via SMS text messaging or email may improve the adherence to web-based programs and increase the probability of successful behavior change; however, it is unclear whether their efficiency is affected by the modality of the communication channel.

**Objective:**

A 2-armed randomized control trial was conducted to compare the effect of providing reminders via SMS text messaging versus email on the adherence to an eHealth program for smoking cessation and on the probability to initiate a quit attempt.

**Methods:**

Smokers were recruited via an internet-based advertisement. A total of 591 participants who diverted from intended use of the program (ie, failed to log on to a session) were automatically randomized to the experimental (SMS text messaging reminder, n=304) or the active comparator (email reminder, n=287) group.

**Results:**

Unexpectedly, we found that the mode of reminder delivery did not significantly affect either the adherence, namely the number of completed program sessions, with the SMS text messaging reminder group showing a mean of 4.30 (SD 3.24) and the email reminder group showing a mean of 4.36 (SD 3.27) (t_586_=0.197, *P*=.84, and Cohen *d*=0.016), or the outcome, namely the quit smoking attempt rate (34.2% in the SMS text messaging group vs 31.7% in the email group; *χ^2^*_1_=0.4, *P*=.52). Secondary analyses showed that age, gender, and education had significant effects on program adherence and education on the outcome. Moreover, we found a significant interaction effect between the mode of reminder delivery and gender on program adherence, suggesting that the effectiveness of SMS text message reminders might be different for females and males. However, this particular finding should be treated with care as it was based on post hoc subgroup analysis.

**Conclusions:**

This study indicates that the modality of user reminders to log on increased neither the program adherence nor the probability of quitting smoking. This suggests that program developers may save costs using emails instead of SMS text messaging reminders.

**Trial Registration:**

ClinicalTrials.gov NCT03276767; https://clinicaltrials.gov/ct2/show/ NCT03276767

## Introduction

Interventions delivered through the internet may provide people with tailored and real-time suggestions [1] and allow targeting and attracting large populations [2-4]. Web-based interventions can help people change health behavior [5], including quitting tobacco smoking [6,7], which is still one of the leading causes of avoidable mortality and morbidity worldwide [8,9]. Although we know that web-based interventions for smoking cessation may improve quitting rates, there is a need for research into factors that may increase the efficacy of such interventions [10].

The efficacy of web-based interventions is closely associated with users’ adherence to them [11], making it pertinent for program designers to find ways of increasing adherence to the programs they design. One way of increasing program adherence is through digital triggers that are external stimuli “designed to make an individual focus on a desired goal by prompting an internal or external reaction at the appropriate time” [12]. Such triggers can be integrated in an otherwise web-based program as notifications when new program content is made available and as reminders for logging on once the user fails to log on as expected. However, developing digital triggers involves a range of design choices, including “who” (ie, sender), “how” (ie, medium), “when” (ie, triggered by what), “how much” (ie, how often), and “what” (ie, content) [12]. Optimizing these design choices in the best manner is an empirical question; however, the evidence on how to design effective triggers is mixed due to insufficient reporting of design choices and heterogeneity in studies [12]. This study seeks to contribute to this knowledge base by specifying the best choice of the delivery mode (the “how”) for digital triggers designed to increase adherence of users who fail to log on as expected. Different options exist, but 2 commonly used alternatives are SMS text messaging and emails, with each having different advantages and disadvantages [12]. For example, SMS text messaging (compared to email) is more salient to the receiver [3] and linked to higher open and click rates [12], but there is a higher cost associated with SMS text messaging (ie, the cost of sending as well as that for development and maintenance of the system). A meta-study of web-based interventions found that using additional methods of communicating with participants, like email or SMS text messaging, were associated with larger effects on behavior change, and more specifically, this effect was reported to be large for SMS text messaging and small for email [10]. However, a limitation of this meta-study is that these conclusions were based on comparing the effect sizes in studies with no reminders or email reminders to studies using SMS text messaging reminders. Several direct comparisons of modalities across arms within a randomized trial, and across various contexts, is needed to address this issue. Nevertheless, these studies appear to indicate that regarding reminders to log on to a web-based program, SMS text messaging reminders would be superior to email reminders. Addressing this matter is significant for program designers, as adding an SMS text messaging component will usually entail additional costs to program development and should thus be worth the money.

Therefore, the purpose of this study was to determine whether SMS text messaging would indeed be more effective in reminding the users to log onto the web application once they fail to log on as expected. Our point of departure was a web-based smoking cessation program (described below) that uses email invitations to notify the user of the release of new program modules. If the user does not log on as expected, the program will send out a reminder to prompt the user to log on. We conducted a randomized controlled trial (RCT) in which participants were randomized to receive these reminders either by SMS text messaging or email. We hypothesized that SMS text messaging reminders would have users logging onto the web application frequently (ie, adherence) and possibly lead to an increased likelihood of an initiated attempt to quit smoking (due to increased program use).

## Methods

### Study Design

A 2-armed RCT was conducted. The 2 arms of the RCT differed only in the modality (SMS text messaging versus email) of the reminders that were issued to remind the smoker about the missed session (module). There were 4 different versions of this message, so that each user would never receive the same message twice in a row. The messages were very similar in content and form, for example, “Hi (name of user) I haven’t seen you for a while. You may have been busy? Hope to see you soon! (smart link to the web application) Best Andy (Andy is the English name of the intervention).” The study protocol was registered at ClinicalTrials.gov (trial registration number: NCT03276767).

### Participants

**Figure 1:**
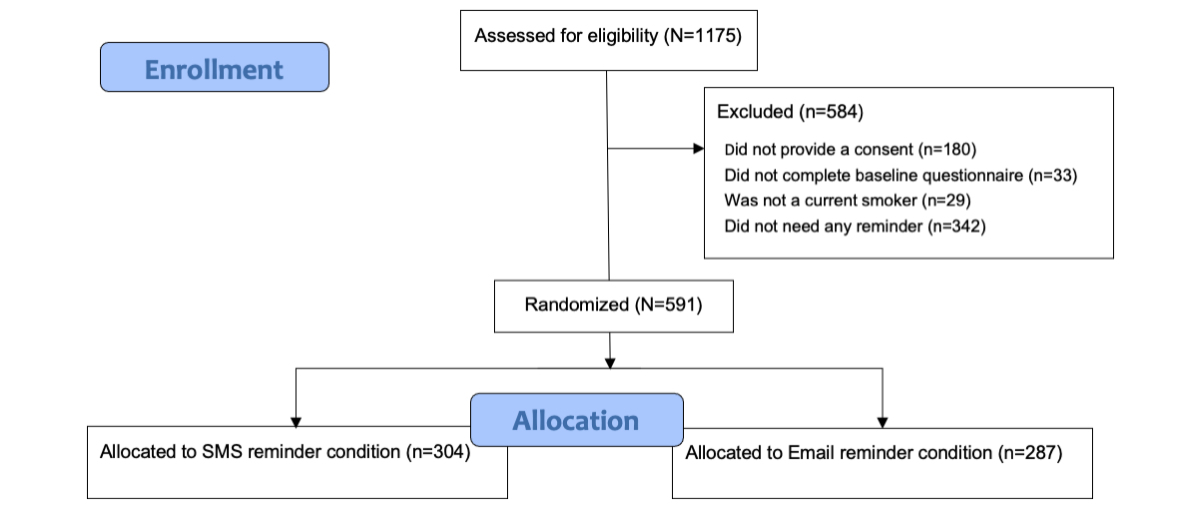
Flow diagram of participant selection.

The study sample consisted of Czech and Norwegian tobacco smokers using Andy, an eHealth smoking cessation program (described below). Czech participants were recruited through internet-based advertisements (webpages focused on smoking cessation, social media platforms, and internet-based newspapers). Norwegian participants were recruited through internet-based advertisements (Facebook, Google, blogposts, and newspapers) as well as through Healthy Life Centers. Participants needed to be over 18 years old, current tobacco smokers, willing to quit smoking, provide a valid email address and mobile phone number, provide consent to participation, complete the baseline questionnaire, and open the first session of the program. Additionally, only persons who qualified as nonadherers were included; nonadherence was defined as failing to log on to the program by noon on the day after a new session (module) was released. Overall, 584 of the 1175 recruited participants (49.7%) did not meet the inclusion criteria, resulting in a total of 591 participants. Figure 1 shows the participant selection process.

### Data Collection

Baseline characteristics were collected through a web-based questionnaire, whereas program use information was recorded automatically. Program use information included the start and completion of a session, issuing of SMS text messaging/email reminders, and user-reported initiation of a quit attempt.

### Intervention and Randomization

The eHealth intervention named “Andy” is a fully automated web-based smoking cessation program that first prepares the user for quitting (preparation phase) and follows up once the user confirms having initiated a quit attempt (follow-up phase); however, this study focuses only on the preparation phase. During the preparation phase, the program releases 1 new session per day. The recommended/default length of the preparation phase is 11 days (which is the designated quit day), but users are free to advance or postpone at their own rate within certain limits. When a new session is released, an email invitation is sent to the user to prompt login (this happens at 5 AM). A reminder is due when a user fails to log onto an assigned session by noon on the next day. In this study, a simple randomization procedure automatically took place when the first reminder was due, following which participants were randomized to either the SMS text messaging or the email condition. More information about the intervention program, how it is used, and its usability can be obtained elsewhere [13-16].

### Measures

#### Demographics

Participants were asked about their age, gender, place of residence (urban or rural area), education, employment, and income.

#### Baseline Smoking

Participant also reported their average consumption of cigarettes (self-reported number of cigarettes smoked per day). Following a standard procedure [17], we then categorized participants as a mild (<10 cigarettes/day), moderate (11-19 cigarettes/day), or intensive (>20 cigarettes/day) smokers.

#### Primary Outcome: Initiation of Quit Attempt

The main outcome was whether the participants had initiated a quit attempt. Upon logging on to the program on the quit day, participants are asked whether they have in fact initiated a quit attempt as planned. Those who answer “no” are encouraged to quit the next day and will be asked the same question the day after (and on any subsequent day if they keep logging on to the program). Those who answer “yes” will be transferred to the follow-up phase of the program. Participants who dropped out from the program before they reached their quit day were considered treatment failures (ie, smokers). Participants who continued to log on to the program until their quit day (or beyond) but did not report to have initiated a quit attempt within 6 weeks after accessing the first session of the program were also considered treatment failures.

#### Secondary Outcome: Number of Completed Sessions

Program adherence was measured as the number of eHealth program sessions that participant had completed. It was possible to complete 0 to 10 sessions during the preparation phase of the eHealth program. A higher number of completed sessions indicated higher adherence to the eHealth program.

### Statistical Analysis

Statistical analyses were conducted in multiple steps. First, we aimed to assess randomization by confirming the absence of significant differences in background variables between the experimental group (SMS text messaging reminders) and the active control group (email reminders). Second, we used the Welch 2-tailed *t* test for unequal variance to analyze the effect of SMS text messaging reminders on program adherence, namely the number of completed program sessions (secondary outcome). Next, we used the *χ^2^* test of association for assessing the effect of SMS text messaging reminders on the initiation of a quit attempt (primary outcome). Moreover, 2 regression analyses were conducted to assess the effects of SMS text messaging reminders when controlling for the effects of all background variables (shown in Table 1) on the primary and secondary outcomes. Post hoc analyses of the relationship between the background and outcome variables were also conducted. Analyses were conducted with the statistical software R [18,19].

### Ethics

The study was approved by the Ethics Committee of the General University Hospital in Prague (no. 7/17GrantGACR-1.LFUK) and by the Norwegian Center for Research Data (no. 52874).

## Results

### Baseline Characteristics

Background characteristics of each group are reported in Table 1. Participants (program users) had a mean age of 39.5 (SD 12.8) years, and 52% of the 591 participants (n=308) were Czech and 48% (n=288) were Norwegian. Additionally, 61% (n=361) were female, 60% (n=355) were full-time employees, and 56% (n=330) reported high school as their highest completed education. The mean consumption of tobacco among participants at baseline was 18 cigarettes per day, and 43% (n=254) reported consuming more than 20 cigarettes per day. There were no statistically significant differences found between the SMS text messaging and email groups, except for the nationality distribution (Table 1).

**Table 1:** Baseline characteristics of the experimental (SMS text messaging) group and active control (email) arm of the randomized controlled trial (N=591)^a^.

Characteristic		SMS text messaging (n=304, 51.4%)	Email (n=287, 48.6%)	*t*/*χ^2^*(*df*)	
Age in years (range 18-77), mean (SD)		40.0 (12.9)	38.8 (12.8)	*t* (587)=1.12	
**Nationality, n (%)**					*χ^2^* (1)=8.3
	Czech	141 (45.8)	167 (54.2)		
	Norwegian	163 (57.6)	120 (42.4)		
**Gender, n (%)**					*χ^2^* (1)=0.5
	Female	190 (52.6)	171 (47.4)		
	Male	114 (49.6)	116 (50.4)		
**Residence, n (%)**					*χ^2^* (3)=3.8
	<1000 inhabitants	47 (46.1)	55 (53.9)		
	1000-20,000 inhabitants	94 (57)	71 (43)		
	20,000-100,000 inhabitants	92 (52)	85 (48)		
	>100,000 inhabitants	71 (48.3)	76 (51.7)		
**Education, n (%)**					*χ^2^* (3)=1.7
	<HS^b^ graduate	26 (47.3)	29 (52.7)		
	HS graduate	169 (51.2)	161 (48.8)		
	University (BA^c^ degree)	73 (55.7)	58 (44.3)		
	University (MA^d^ degree or higher)	36 (48)	39 (52)		
**Employment, n (%)**					*χ^2^* (5)=1.0
	Freelancer	35 (53)	31 (47)		
	Employed	178 (50.1)	177 (49.9)		
	Unemployed	41 (55.4)	33 (44.6)		
	Student	25 (50)	25 (50)		
	Retired	11 (52.4)	10 (47.6)		
	Other	14 (56)	11 (44)		
**Income, n (%)**					*χ^2^* (4)=7.4
	Very low	41 (56.9)	31 (43.1)		
	Low	82 (59.4)	56 (40.6)		
	Middle	58 (45)	71 (55)		
	High	92 (49.2)	95 (50.8)		
	Very high	19 (46.3)	22 (53.7)		
**Smoking, n (%)**					*χ^2^* (2)=0.1
	<10 cigarettes/day	79 (52.3)	72 (47.7)		
	11-19 cigarettes/day	96 (51.6)	90 (48.4)		
	>20 cigarettes/day	129 (50.8)	125 (49.2)		
Reminders (range 1-8)		2.58 (1.17)	2.56 (1.09)	*t* (589)=0.27	

^a^*P* values for all variables were not significant except Nationality (*P*<.001).

^b^HS: high school.

^c^BA: Bachelor of Arts.

^d^MA: Master of Arts.

### Effectiveness of SMS Text Messaging Versus Email Reminders on Program Adherence – The Number of Completed Sessions (Secondary Outcome)

The number of completed sessions among all participants ranged from 0 to 10 (mean 4.33, SD 3.26). Contrary to our expectations, we did not find any statistically significant difference in the number of completed sessions between participants receiving SMS text messaging reminders (mean 4.30, SD 3.24) and those receiving email reminders (mean 4.36, SD 3.27); Welch *t*_586_=0.197, *P*=.84, and Cohen *d*=0.02. Given the difference in the Czech/Norwegian participant ratio between the SMS text messaging and email groups, we conducted a regression analysis where we controlled for nationality (and other background variables) with the same result. Receiving SMS text messaging reminders (as compared with receiving email reminders) did not lead to a significantly higher number of completed sessions (*P*=.98). Table 2 presents the regression analysis results.

Some background characteristics were found to be significant predictors of the number of completed sessions, namely age, nationality, gender, and education. Using separate analyses for each of these predictors, we found that higher age was positively associated with adherence (*r*=0.17, *P*<.001) and female participants showed a significantly higher number of completed sessions (mean 4.64, SD 3.32) compared to male participants (mean 3.84, SD 3.09); Welch *t*_513_=2.98, *P*=.003, and Cohen *d*=0.25. Furthermore, participants with a university degree, namely Bachelor of Arts or Master of Arts, completed more sessions compared to those without high school graduation; the omnibus difference was significant (*F*_3,166_=3.87, *P*=.01) with Games-Howell post hoc *t* tests proving significant for differences between those without high school graduation and those with BA (*P*=.02) and MA (or higher) degrees (*P*=.02). The difference in the number of completed sessions between Czech (mean 4.17, SD 3.14) and Norwegian (mean 4.51, SD 3.37) participants was not significant (*P*=.27).

**Figure 2:**
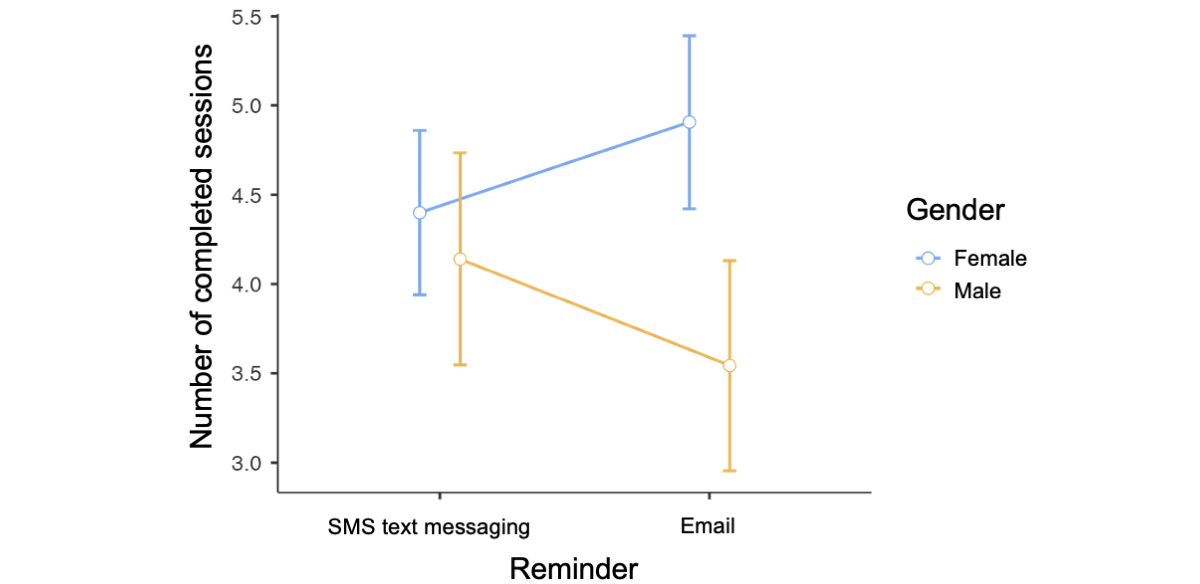
Effects of randomized condition (SMS text messaging versus email reminders) and gender on the electronic health program adherence (the number of completed sessions). Estimated marginal means with 95% CIs are shown.

Although we found no significant main effect of the reminder delivery mode on adherence, post hoc analyses revealed a significant interaction with gender; *F*_1,587_=4.10 and *P*=.04. The finding suggests that the effect of replacing email reminders with SMS text messaging reminders is more beneficial for males, relative to females, in terms of improved program adherence. The average number of completed sessions for female users receiving SMS text messaging reminders (n=190) was 4.40 (SD 3.40), whereas for male users receiving SMS text messaging reminders (n=114), it was 4.14 (SD 2.98); further, for female users receiving email reminders (n=171), the average number of completed sessions was 4.91 (SD 3.22), and for male users receiving email reminders (n=116), it was 3.54 (SD 3.19) (Figure 2).

**Table 2:** Summary of linear regression analysis for variables predicting the number of completed sessions (N=591, R^2^=0.0796)^a^.

Predictor		Regression analysis variables						
		*B*		SE *B*	*t*	*P*	β	95% CI
**Reminder**								
	SMS text messaging		0.007	0.2725	0.025	.98	.002	–0.162 to 0.166
Age			0.052	0.0158	3.288	.001	.200	0.081 to 0.320
**Nationality**								
	Czech		0.951	0.4608	2.064	.04	.292	0.014 to 0.569
**Gender**								
	Female		0.771	0.3187	2.419	.02	.236	0.044 to 0.428
**Residence**								
	1000-20,000 inhabitants		0.195	0.4208	0.464	.64	.060	–0.194 to 0.313
	20,000-100,000 inhabitants		–0.200	0.4133	–0.485	.63	–.061	–0.310 to 0.187
	>100,000 inhabitants		–0.373	0.4455	–0.838	.4	–0.114	–0.383 to 0.154
**Education**								
	HS^b^ graduate		0.846	0.5069	1.668	.1	.259	–0.046 to 0.565
	University (BA^c^ degree)		1.197	0.5763	2.078	.04	.367	0.020 to 0.714
	University (MA^d^ degree or higher)		1.218	0.6370	1.912	.06	.373	–0.010 to 0.757
**Employment**								
	Employed		0.537	0.4558	1.178	.24	.165	–0.110 to 0.439
	Unemployed		–0.231	0.6446	–0.358	.72	–.071	–0.459 to 0.317
	Student		–0.458	0.7891	–0.580	.56	–.140	–0.616 to 0.335
	Retired		–0.170	0.9076	–0.187	.85	–.052	–0.600 to 0.500
	Other		–1.091	0.8291	–1.316	.19	–.334	–0.834 to 0.165
**Income**								
	Low		–0.580	0.5079	–1.142	.25	–.178	–0.484 to 0.128
	Middle		–0.286	0.5792	–0.494	.62	–.088	–0.440 to 0.261
	High		–0.492	0.6555	–0.751	.45	–.151	–0.546 to 0.244
	Very high		–1.031	0.8109	–1.272	.2	–.316	–0.805 to 0.172
**Smoking**								
	11-19 cigarettes/day		0.068	0.3691	0.185	.85	.021	–0.201 to 0.243
	>20 cigarettes/day		–0.260	0.3643	–0.713	.48	–.080	–0.300 to 0.140

^a^*B* represents the log odds of quit attempt=1 versus quit attempt. represents standardized estimates. “Email” is the reference category for Reminder. “Norwegian” is the reference category for Nationality. “Female” is the reference category for Gender. “<1000 inhabitants” is the reference category for Residence. “<HS graduate” is the reference category for Education. “Freelancer” is the reference category for Employment. “Very low” is the reference category for Income. “<10 cigarettes/day” is the reference category for Smoking.

^b^HS: high school.

^c^BA: Bachelor of Arts.

^d^MA: Master of Arts.

### Effectiveness of the SMS Text Messaging and Email Reminders on the Initiation of Quit Attempt (Primary Outcome)

In the whole sample comprising 591 participants, 195 (33%) participants initiated a quit attempt. Contrary to our hypothesis, there was no significant difference between the 2 randomized groups in initiating a quit attempt (*χ^2^*_1_=0.4, *P*=.52). The frequency of quit attempts in the SMS text messaging group was 104 (34.2%) whereas it was 91 (31.7%) in the email group. We did not find any significant interaction between education, receiving SMS text message versus email reminders, and quit attempts. Regression analysis in which background variables were controlled for also showed that receiving SMS text messaging reminders instead of email reminders was not a significant predictor of initiating a quit attempt (Table 3). From all analyzed sociodemographic variables, only education was a significant predictor of initiating a quit attempt (Table 3). Participants with an education lower than high school (ie, elementary or practical education) reported initiating a quit attempt in 9 (16.4%) cases, showing a 2 times lower prevalence than in the whole sample. In comparison, 106 (32.1%) of the high school–graduated participants, 49 (37.4%) of the college-graduated participants with a bachelor’s degree, and 31 (41.3%) of the college-graduated participants with a master’s degree reported having initiated an attempt to quit smoking (*χ^2^*_3_=10.5, *P*=.02).

**Table 3:** Summary of logistic regression analysis for variables predicting quit attempt in electronic health program users (N=591, R^2^=0.034)^a^.

Predictor		Regression analysis variables				
		*B*	SE *B*	Z^b^	*P*	Odds ratio, 95% CI
**Reminder**						
	SMS text messaging	0.1394	0.1852	0.7530	.45	1.150, 0.7997-1.653
Age		0.0163	0.0107	1.5273	.13	1.016, 0.9954-1.038
**Nationality**						
	Czech	0.1477	0.3152	0.4686	.64	1.159, 0.6250-2.150
**Gender**						
	Female	–0.0136	0.2153	–0.0632	.95	0.986, 0.6468-1.504
**Residence**						
	1000-20,000 inhabitants	–0.0909	0.2843	–0.3197	.75	0.913, 0.5231-1.594
	20,000-100,000 inhabitants	–0.1642	0.2795	–0.5874	.56	0.849, 0.4906-1.468
	>100,000 inhabitants	–0.1370	0.3010	–0.4553	.65	0.872, 0.4834-1.573
**Education**						
	HS^c^ graduate	0.8912	0.4158	2.1433	.03	2.438, 1.0792-5.508
	University (BA^d^ degree)	0.9175	0.4542	2.0202	.04	2.503, 1.0277-6.096
	University (MA^e^ degree or higher)	1.1533	0.4855	2.3755	.02	3.169, 1.2235-8.206
**Employment**						
	Employed	0.2879	0.3168	0.9087	.36	1.334, 0.7167-2.482
	Unemployed	0.0998	0.4419	0.2259	.82	1.105, 0.4647-2.627
	Student	0.0595	0.5551	0.1072	.92	1.061, 0.3576-3.150
	Retired	–0.5661	0.6345	–0.8923	.37	0.568, 0.1637-1.969
	Other	–0.3773	0.6125	–0.6160	.54	0.686, 0.2065-2.278
**Income**						
	Low	–0.1251	0.3522	–0.3552	.72	0.882, 0.4425-1.760
	Middle	0.1129	0.3958	0.2851	.78	1.119, 0.5154-2.432
	High	–0.0173	0.4484	–0.0386	.97	0.983, 0.4081-2.367
	Very high	–0.5921	0.5684	–1.0418	.3	0.553, 0.1816-1.685
**Smoking**						
	11-19 cigarettes/day	0.1315	0.2429	0.5414	.59	1.141, 0.7085-1.836
	>20 cigarettes/day	–0.3045	0.2475	–1.2304	.22	0.737, 0.4540-1.198

^a^*B* represents the log odds of quit attempt=1 versus quit attempt. “Email” is the reference category for Reminder. “Norwegian” is the reference category for Nationality. “Female” is the reference category for Gender. “<1000 inhabitants” is the reference category for Residence. “<HS graduate” is the reference category for Education. “Freelancer” is the reference category for Employment. “Very low” is the reference category for Income. “<10 cigarettes/day” is the reference category for Smoking.

^b^Z: regression coefficient divided by the standard error.

^c^HS: high school.

^d^BA: Bachelor of Arts.

^e^MA: Master of Arts.

## Discussion

### Principal Results

This RCT tested the hypothesis that receiving SMS text messaging reminders (compared to receiving email reminders) increased (1) the adherence to the eHealth program for smoking cessation and (2) the initiation of an attempt to quit smoking. Randomization took place after the first sign of nonadherence to the eHealth program (ie, when a user failed to log on to the program as expected and was due to receive the first reminder). There were no significant differences between the 2 groups in terms of their background characteristics, except for nationality. Norwegian participants were more often randomized to the SMS text messaging group as compared to Czech participants. The adherence to the eHealth program was measured as the number of completed sessions and the desired outcome was measured as self-reported initiation of a quit attempt. Surprisingly, we did not find any significant differences in the number of completed sessions between participants receiving SMS text messaging reminders (completed 4.30 sessions on average) and those receiving email reminders (completed 4.36 sessions on average), when tested separately (*P*=.84) or when controlled for all the background variables listed in Table 1 (*P*=.98). Similarly, we did not find differences in the proportion of reported quit attempts between SMS text messaging (quit attempt reported by 34.2% participants) and email (quit attempt reported by 31.7% participants) groups, either when measured separately (*P*=.52) or when controlled for all background variables (*P*=.45).

Additional post hoc analyses revealed significant effects of some sociodemographic variables on program adherence (age, gender, and education) and the initiation of a quit attempt (education). None of these effects interacted significantly with the reminder modality (SMS text messaging versus email), except for gender, which is attributable to the modality interaction effect on program adherence (*P*=.04). In other words, the effect of replacing email reminders with SMS text messaging reminders on program adherence is heterogenous across genders. This effect suggests that SMS text messaging reminders are more beneficial for men, relative to women, in terms of program adherence (see Figure 2). (Note that we did not find any interaction between the modality and gender on quit attempts, or between the modality and any other background variable.)

In summary, post hoc subgroup analyses revealed that the choice of the optimal modality may depend on gender. SMS text messaging is more beneficial for males relative to females regarding program adherence. We did not find overall improvement in program adherence on receiving SMS text messaging reminders when compared to email reminders. More importantly, we found that the reminder modality did not affect the main outcome, namely smoking cessation.

### Comparison With Prior Work

Prior work has shown that external triggers, such as reminders, may improve adherence to eHealth programs and thus the outcomes of these interventions [12]. The evidence of how to design effective triggers is mixed due to insufficient reporting of design choices and heterogeneity in studies [12]. This study focused on the mode of delivery of triggers (reminders), for which SMS text messages and emails are 2 popular options considered by designers. Compared to an email, an SMS text message is more salient to the receiver [3], linked to higher open and click rates [12], and associated with larger effects on eHealth-supported behavioral change [10]. These previous findings suggest that SMS text messaging is superior to email for reminding users to log on to an eHealth web program, thus increasing adherence and the probability of desired outcomes, and is therefore worth the additional cost. However, contrary to our expectations, we did not find evidence supporting this superiority of SMS text messaging over email reminders either with respect to the program adherence or to the outcomes of this specific eHealth program (ie, the initiation of a quit attempt). However, the effect of the reminder delivery mode on program adherence may be affected by gender; in our study, female participants were found to be generally more adherent to the eHealth program (completed more sessions) and the difference was particularly strong in the condition of email reminders. Although there is some evidence that women are more compliant with eHealth interventions in general [20], to our best knowledge, there is no study available that has analyzed the relationships between gender, eHealth adherence, and different modes of delivering reminders. Our results suggested that SMS text messaging reminders (compared to email reminders) might help reduce the gender-based difference in adherence. This would be an interesting area for further research. It should be noted that many other factors might be influencing the efficiency of the reminders, such as content, frequency, time of delivery, type of the intervention program (eg, web-based or mobile app, frequency and number of session releases, etc), or even the phase of the intervention (eg, reminders might affect users differently when received at the beginning as opposed to the later phase).

### Strengths and Limitations

This study has several strengths, including a heterogenous sample with participants belonging to a wide spectrum of sociodemographic groups (Table 1) from the Czech Republic and Norway, 2 countries with different levels of smoking prevalence and tobacco use patterns. The other strength is that the 2 groups differed only in the mode of communication for reminders to log on to the program, whereas the content and number of reminders as well as the content of the program in general were the same for both groups. Therefore, we could assess the direct effect of the reminder delivery mode on program adherence and the desired outcome of the intervention. Generally, SMS text messaging reminders are often used within health care but RCTs assessing their effect are lacking [21]. In addition, the use of automatically collected eHealth data reduced selection bias and the risk of recall bias (although the initiation of a quit attempt was self-reported).

One limitation of the study is that the preparation phase of the program was fairly short (maximum 11 days), resulting in a short period for assessing the adherence. Moreover, the study focused on a smoking cessation eHealth program and may not be generalized for other types of eHealth interventions. Findings concerning the interaction between SMS text messaging reminders and gender are based on post hoc subgroup analysis, and as such, it should be treated with care [22]. Further, a question that our study did not address was that individual differences might influence the effect of the reminder modality. Further research might inquire into the potential of tailoring reminder modalities to individual preferences.

### Conclusions

In conclusion, and contrary to available literature, our data suggested that when it comes to reminding nonadherent eHealth users to log on to a web-based program, SMS text messaging reminders were not superior to email reminders, neither with respect to increasing program adherence nor in supporting a desired outcome (ie, the initiation of a quit attempt). However, there may be gender differences affecting the preferred modality (with email reminders being more effective for female users) that may be useful to pursue in further research. The results for both outcomes taken together suggest that there is very little to gain, if anything at all, by choosing SMS text messaging over email reminders for web-based behavior change interventions. Thus, our finding is important for developers and providers of eHealth interventions who may not need to allocate additional costs related to SMS text messaging reminders to enhance program adherence or outcomes, as reminders delivered via email seem to be equally effective.

## Appendix

Multimedia Appendix 1 CONSORT eHealth checklist (V 1.6.1).

## Abbreviations

RCT: randomized controlled trial
